# Computer Assisted Chronic Disease Management: Does It Work? A Pilot Study Using Mixed Methods

**DOI:** 10.5402/2013/801723

**Published:** 2013-03-07

**Authors:** Kay M. Jones, Ruby Biezen, Leon Piterman

**Affiliations:** ^1^Department of General Practice, Monash University, Building 1, 270 Ferntree Gully Road, Notting Hill, VIC 3168, Australia; ^2^Berwick & Peninsula, 100 Clyde Road, Berwick, VIC 3806, Australia

## Abstract

*Background*. Key factors for the effective chronic disease management (CDM) include the availability of practical and effective computer tools and continuing professional development/education. This study tested the effectiveness of a computer assisted chronic disease management tool, a broadband-based service known as cdmNet in increasing the development of care plans for patients with chronic disease in general practice. *Methodology*. Mixed methods are the breakthrough series methodology (workshops and plan-do-study-act cycles) and semistructured interviews. *Results*. Throughout the intervention period a pattern emerged suggesting GPs use of cdmNet initially increased, then plateaued practice nurses' and practice managers' roles expanded as they became more involved in using cdmNet. Seven main messages emerged from the GP interviews. *Discussion*. The overall use of cdmNet by participating GPs varied from “no change” to “significant change and developing many the GPMPs (general practice management plans) using cdmNet.” The variation may be due to several factors, not the least, allowing GPs adequate time to familiarise themselves with the software and recognising the benefit of the team approach. *Conclusion*. The breakthrough series methodology facilitated upskilling GPs' management of patients diagnosed with a chronic disease and learning how to use the broadband-based service cdmNet.

## 1. Introduction

Chronic diseases are a major health concern and major cost in Australia [1] and in the most developed and developing countries. Since 1999, the Australian government, through its national health insurance scheme known as Medicare, has sequentially introduced a range of incentives including subsidised computers, payments to GPs via the Medicare schedule for chronic disease management (CDM) [2] including general practice management plans (GPMPs, item 721), team care arrangements (TCAs, item 723), reviews of GPMPs and/or TCAs, home medicines reviews (HMR, item 900) [2], and items for work undertaken by general practice nurses on behalf of a GP including administration of these care plans [3–5]. As in November 2012, according to the Medicare schedule fees (for payment to GPs) Reimbursement for these items was: Item 721 $141.40, Item 723: $112.05, item 732: $70.65, and item 900: $151.75 [2].

Several key factors have been identified for effective chronic disease management including: the availability and accessibility of information technology (IT), accessibility of patients' clinical information, patient/consumer participation in decision making, good linkages with community resources and services, longer consultations for patients with GPs, GPs delegating roles and responsibilities to other health professionals, and using models of care [6–9]. While Medicare data suggests that less than 14% of patients with a chronic disease have a GPMP and/or a TCA, only one in five of these plans is regularly followed up and reviewed at the recommended frequency [10].

Effective continuing professional development is also of importance for quality improvement in clinical practice [11, 12]. The breakthrough series methodology which includes a model for improvement (plan-do-study-act) [13] has been described as a strategy for improving quality outcomes in general practice in Australia and has been used to upskilled GPs in chronic disease management [14, 15]. 

This mixed methods study tested a broadband-based service known as cdmNet, which creates web-based GPMPs using disease specific templates by extracting and auto-populating data from GPs' computer records into the GPMP and sharing this with designated member of the care team [14–17]. cdmNet was developed and is managed by an Australian-based external provider, Precedence Health Care (PHC).

Comprising workshops and interviews, this study aimed to promote best practice in GP management of patients diagnosed with a chronic disease, in particular, using Medicare Item numbers 721, 723, 732, and 900 and cdmNet. While this study was conducted in Australia, the impact of the introduction of information technology is of interest globally, where information technology is being introduced for chronic disease management.

## 2. Methodology

### 2.1. Mixed Methods

1. The breakthrough series methodology including the plan-do-study-act model [13–15], comprising of four learning workshops and a followup workshop over a period of 8 months;
2. semistructured interviews with GPs who participated in the learning workshops [18, 19].

### 2.2. Recruitment

Regarding learning workshops, a convenience sample of GPs was recruited through the Monash University, Department of General Practice networks. The letter of invitation was circulated to 508 GPs, of those, 57 GPs expressed interest; 24 GPs, 2 practice staff and 6 research staff, attended the first learning workshop.

Regarding interviews, of the 24 GPs who commenced workshop series, 15 agreed to participate. GPs were invited to participate in the interviews during learning workshop 2; a followup telephone call was made to (a) confirm agreement to participate and (b) to arrange a time to conduct the interview. A semistructured interview schedule was developed based on the literature (Box 1).

### 2.3. Data Collection

Data were collected using three tools specifically developed for this study:

1. a predisposing activity completed two weeks before each learning workshop,
2. an evaluation completed during each learning workshop,
3. a reinforcing activity completed two weeks after each learning workshop.

Interviews were conducted during August-September 2011 at the participating GPs' practices. 

### 2.4. Data Analysis

Qualitative data were analysed using content analysis [18, 19]; quantitative data were analysed using SPSS v.19 [20].

Ethics for the project were submitted and approved by Monash University Human Research Ethics Committee (MUHREC).

## 3. Results

### 3.1. Workshops

Throughout the intervention period (from March to November 2011) and the period up until the followup workshop (November 2011), GPs were asked to report the number of GPMPs (721), TCAs (723), reviews (732), and HMRs (900) created. During this time, the number of items created and completed initially increased, reduced, and then increased again (Table 1). GPs felt this peak and plateau similarly reflected that they were managing their “usual” throughput after managing their backlog.

GPs were also asked to report the number of item 721 that they had created for four chronic diseases: diabetes, osteoarthritis, CHD/CVD, and COAD/COPD during the intervention period (Table 2). A similar pattern emerged with the number of Item 721′s developed; increasing then plateauing after workshop 2-3. Discussion during workshops indicated that this may have occurred because GPs felt they had identified most of their patients who they had not previously considered for a GPMP, and then after the backlog had been addressed, the numbers reported at later workshops were more reflective of their “usual” throughput.

In addition to the four chronic diseases (Table 2), GPs created GPMPs and TCAs conducted reviews and ordered HMRs for other chronic diseases including depression, asthma, chronic low back pain, chronic kidney disease, stroke, and type 1 diabetes (Table 1). 

**Figure 1:**
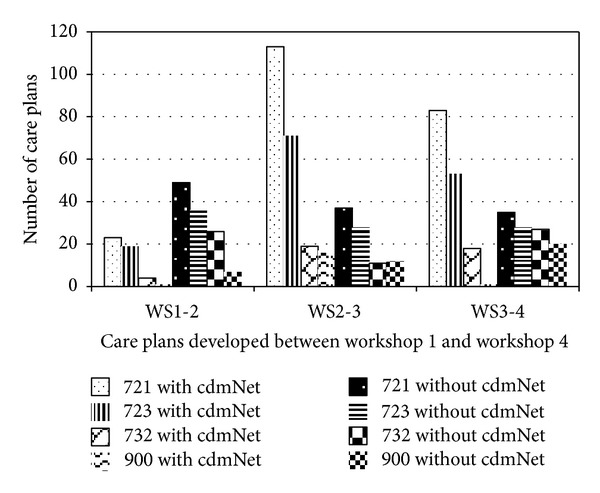
Care planning activity “using” and “not using” cdmNet throughout the project.

Regarding the use of cdmNet, GPs were asked to estimate any shift from “not using” to “using” cdmNet during the intervention period (Figure 1). For GPMPs (Item 721), while there was an initial increase then a reduction of “using” cdmNet, there was a small but noticeable reduction in the number of GPMPs developed “not using” cdmNet. A similar pattern emerged for TCAs (item 723) “using” cdmNet, but the number developed “not using” cdmNet plateaued during the latter part of the intervention period. It was suggested that this occurred because GPs were developing TCAs for GPMPs that they had previously developed not using cdmNet. Regarding reviews (item 732) and HMRs (item 900) that the increase and plateau “using cdmNet” and the reduction and increase “not” using cdmNet, it was felt that these patterns reflected the followup patterns for GPMPs and TCAs developed prior to the introduction of cdmNet. 

Thus, a pattern was emerging suggesting an increase in the use of cdmNet for GPMPs and TCAs, but not for reviews and HMRs during the intervention period; however, it was felt that the pattern for reviews and HMRs would follow the pattern for GPMPs and TCAs in the future. This also suggests that an increase in the number of care planning items developed by GPs using cdmNet may contribute to increase GP's income.

GPs also spoke about changes in their practice nurses' and practice managers' roles. Practice nurses were becoming more involved in generating GPMPs using cdmNet, identifying eligible patients, spending more time completing health assessments and preparing GPMPs and reviews. Practice managers were becoming more involved in supporting GPs by creating “eligible patients” lists, assisting in coordination, entering data on the computer, and identifying patients for recalls and reviews. 

Throughout the intervention period, GPs evaluated the learning workshops as being of benefit. Benefits and the value of the workshops reported by GPs included the following:

1. learning from other GPs that they are also experiencing similar challenges, in particular, the low interest of specialists in management plans whether they are Internet generated or not,
2. resolving how to add lists of allied health providers to cdmNet,
3. identifying the difference between lack of details generally recorded in paper-based care planning documents (GPMPs and TCAs) compared with the detail recorded in a cdmNet-generated plan,
4. the ease at which cdmNet works compared to the initial perception that cdmNet is complex,
5. the (reduced) time required to modify documents to meet individuals' needs, when using cdmNet,
6. an awareness that cdmNet is still an evolving process that requires IT infrastructure and information sharing across the health system.

### 3.2. GP Interviews

Seven main messages emerged.

*(1) Learning the Program.* Having computers available at the workshop for GPs to practice was valued, as was the medico-legal session. Other benefits included learning from each other about how to use cdmNet, which among other things, contributed to GPs being more aware of patients' eligibility for GPMPs, TCAs, reviews, and HMRs:

*“… practice makes perfect-by, using the information obtained from the education activity workshops and using the knowledge straight away” (GP1).*

*(2) Implementation of cdmNet.* For some GPs, implementation of cdmNet was delayed for reasons beyond the project team's control; thus, GPs were at varying stages of learning the information presented, and subsequently discussion at workshops did not always reflect the stages for all GPs:

*“… we were a bit late getting it loaded on because we had some software issues and other things … we started doing a few things ourselves so we were getting familiar with the format and process and that sort of thing, it all made a bit of sense … and at the education meetings they were trying to troubleshoot and ease the concerns and help us feel comfortable and answer questions about use and that sort of thing, so that was all fine” (GP3).*

*(3) Time.* For most, learning the system took more time than initially anticipated. Being “time poor”, GPs reported that their lack of time meant they did not have the opportunity or the “time” to create GPMPs for patients:

*“… yeah, the more complex it is, when you're under pressure already running late, you think, no I cannot do this today” (GP 15).*

*(4) Allied Health Providers and Specialists.* For many, uploading allied health providers' and specialists' information onto the “address book” was an issue. Although PHC staff provided advice and assisted GPs with this process, there were significant challenges in completing this task:

*“… one of the issues is having huge numbers of names to scroll through to find the ones that we use [in the address book] … so that's probably the major problem” (GP9).*

*(5) Patients and Privacy.* The majority of GPs indicated they had some patients who were concerned with privacy issues and some patients who cannot afford the cost of private AHP/specialists. Generally, the community centres that the GPs in this cohort referred to did not have cdmNet installed in their IT systems:

*“… I had one patient with multiple chronic conditions and I wanted to do a GPMP, but I had to do the old paper system because she is very concerned about privacy and she said she does not have internet, she is one of those persons that is very concerned” (GP2)*

*“… most of the Allied Health people are at the “local” community centre and I suppose it's kind of made me realise in some ways this whole system does not work very well for my patients because most of them do not use private Allied Health people” (GP4).*

*(6) IT Issues.* Identified information technology (IT) issues variously impacted on the GPs. Some expressed concern that the information developed using cdmNet was not automatically recorded back into their medical software; others queried how cdmNet would affect the practice medical software systems and others queried compatibility: 

*“… cdmNet cannot be installed for all GPs because cdmNet is not compatible with some medical software” (GP7).*

*(7) Overall View of cdmNet and Chronic Disease Management.* Overall, GPs felt that using cdmNet raised their awareness of the number of patients with a diagnosis of chronic disease, resulting in more recognition of patients' eligibility and subsequent increase in developing GPMPs. Generally, GPs felt they had become more proactive in developing GPMPs, TCAs, and reviews for patients with chronic disease(s), rather than being “reactive” as they previously had been.

*“… the thing is, as you get more and more familiar; if I get really good on it and I could do them five or six, it would be fantastic” (GP2).*

All agreed that the Internet is seen to be the way of the future. With this in mind, the future use of cdmNet may depend on several factors such as the efficiency of the system, cost benefits for GPs and practices, and better health outcomes for patients.

## 4. Discussion 

This educational intervention achieved its aim. The use of the breakthrough series approach provided significant insight into GPs' management of patients with a chronic disease, in particular, when using Medicare items 721, 723, 732, and 900 and the broadband-based service, cdmNet. 

At each of the workshops, GPs provided feedback about cdmNet which was of value to the developers to inform updating and further development of the broadband-based service. Despite an initial lack of satisfaction with cdmNet, those who completed the GP education workshops generally reported satisfaction with many aspects of cdmNet. The eight GPs who withdrew during the intervention period cited a range of reasons including time commitments, incompatibility with their practice medical IT systems, and challenges with cdmNet. 

Overall changes made by participating GPs regarding their use of cdmNet varied from “no change/having developed no GPMPs using cdmNet” to “change/developing many GPMPs using cdmNet”. While the variation is significant, this may be due to many reasons, not the least, the timing of when cdmNet was installed for participating GPs during the intervention period. 

Feedback from the interviews with the GPs reflected the comments made during the GP education workshops: time to learn the process was required, the uploading of the address book was a challenge for many because of allied health professionals, and specialists could not be included in GPMPs and/or TCAs if their information was not available in the address book. Similarly, not all community centres had cdmNet installed on their IT systems. Other IT issues were also discussed for exploration by PHC, the for example, the incompatibility of cdmNet with some medical IT systems. Nonetheless, GPs generally agreed that using cdmNet raised their awareness about their patients, that GP education workshops prompted them to think about chronic disease management as a process that included a team, and that the Internet is seen to be the way of the future.

The GPs who ceased using cdmNet either during or after completing the GP education workshops were influenced by several issues including time, cost and/or staff (including GPs) unwilling to use the tool. While some felt the concept was good, they did not feel it was of benefit in their practice. 

The strengths of this study include the fact that the education workshops were undertaken in as close to “real life” as possible. GPs indicated that they benefited from, among other things, collegiality, sharing of ideas, and identifying challenges. The data collection tools were developed to ensure that the GPs had a record of the changes they had made throughout the intervention. Interest in the study was strong, and significantly two-thirds completed the GP education workshops. Most participating GPs participated in face-to-face interviews and assisted with circulating questionnaires to patients who had GPMPs developed during the intervention period.

GPs indicated that they felt that cdmNet could be sustained but would be enhanced if broadened to include other templates such as “disability care planning” and “pain management”. Whilst the technology was seen as a potential challenge for some GPs and patients, others felt that cdmNet could be sustained and enhanced by including a wider range of templates for diseases other than chronic disease.

Limitations were also noted. Not all participating GPs had cdmNet installed at the same time. With a difference of several months between the first and final installation, no comparison could be made between GPs' progress regarding use of cdmNet and the Medicare item numbers. In addition, not all GPs who commenced in the study completed all workshops; thus, the data is reduced. 

Generalisability may be challenged by current and future technology regarding, for example, compatibility of cdmNet with medical software and/or adding providers into the address book, what happens in the general practice (general practice routine), and/or staff in the practice who are able/willing to be involved with the technology. Similarly, transferability may depend on what happens in health professionals' practices (GPs, allied health professionals, and specialists), particularly whether health professionals choose to use cdmNet or not.

## 5. Conclusion

During the intervention period, all GPs developed GPMPs and most developed TCAs, with output for some increasing in the earlier part of the intervention period then levelling towards the end of the intervention period. 

The breakthrough series methodology facilitated upskilling GPs' management of patients diagnosed with a chronic disease, in particular, the use of Medicare item numbers 721, 723, 732, and 900 and the application of broadband-based service cdmNet as an enabler to achieve this.

Future work may include the study being replicated with a larger study sample, randomization of participants, and conducting quantitative analysis of the outcomes. The work could include collecting demographics and professional characteristics for those who choose not to participate or commence participation then withdraw.

## Figures and Tables

**Box 1:**
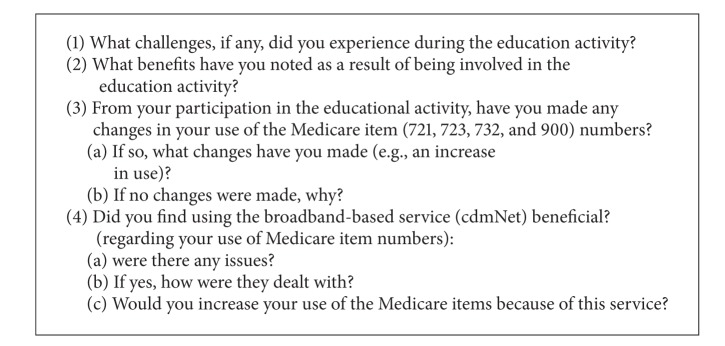
Semistructured interview schedule.

**Table 1:** Care planning activity (721, 723, 732, and 900) from commencement to the completion of project.

	Workshop 1-2	Workshop 2-3	Workshop 3-4	Workshop 4 followup	Total
GPMP (item 721)	57	186	94	110	447
TCA (item 723)	26	109	71	74	280
Reviews (item 732)	8	11	8	17	44
HMR (item 900)	42	91	27	37	197

**Table 2:** Number of 721 for each chronic disease.

Chronic disease	Workshop 1-2	Workshop 2-3	Workshop 3-4	Total
Osteoarthritis	23	83	37	143
Type 2 diabetes	19	65	30	114
CHF/CHD	23	40	15	78
COPD	4	17	6	27
